# Does suture number matter in transvaginal cervical cerclage? A propensity score-weighted cohort study

**DOI:** 10.1007/s00404-026-08444-w

**Published:** 2026-05-02

**Authors:** Edis Kahraman, Nadiye Köroğlu, Turgut Aydın, Mehmet Aytaç Yüksel

**Affiliations:** Department of Obstetrics and Gynecology, Acıbadem Mehmet Ali Aydınlar University, Atakent Hospital, Istanbul, Turkey

**Keywords:** Cervical insufficiency, Emergency cerclage, Prophylactic cerclage, Preterm birth, McDonald technique, Wurm technique

## Abstract

**Purpose:**

To evaluate whether the number of sutures used during transvaginal cervical cerclage influences obstetric and neonatal outcomes when surgical technique, suture material, and perioperative management are standardized.

**Methods:**

This retrospective cohort study included 125 women who underwent transvaginal cervical cerclage using identical monofilament suture material. Cerclage was performed with either a single suture (*n* = 23) or a double suture (*n* = 102) in this non-randomized observational cohort. The primary outcome was term delivery (≥ 37 weeks’ gestation). Secondary outcomes included gestational age at delivery and selected neonatal outcomes. To address non-random allocation and baseline group imbalance, propensity scores were estimated using pre-treatment maternal, obstetric, and clinical severity variables. Stabilized inverse probability of treatment weighting (IPTW) with truncation was applied, and doubly robust outcome models were used to estimate adjusted associations. Neonatal outcomes were analyzed at the pregnancy level to account for clustering in twin gestations.

**Results:**

Women receiving double-suture cerclage presented at earlier gestational ages and with greater markers of cervical severity at baseline; however, after IPTW and doubly robust adjustment accounting for these baseline differences, no statistically significant difference in term delivery was observed. Secondary obstetric outcomes, including gestational age at delivery, were also comparable between groups after adjustment. Pregnancy-level neonatal outcomes, including NICU admission and neonatal mortality, did not differ meaningfully by suture number. Sensitivity analysis restricted to singleton pregnancies and stratified by cerclage indication yielded consistent results.

**Conclusion:**

After accounting for baseline cervical severity and clinical indication, single- and double-suture transvaginal cerclage was associated with comparable obstetric and neonatal outcomes under standardized surgical conditions. These findings suggest that cervical status at the time of cerclage placement, rather than the number of sutures applied, is the primary determinant of outcome.

**Supplementary Information:**

The online version contains supplementary material available at 10.1007/s00404-026-08444-w.

## What does this study add to the clinical work

**Table:** 

The number of sutures used during transvaginal cervical cerclage was not associated with improved obstetric or neonatal outcomes when surgical technique and perioperative management were standardized. Clinical benefit appears to be driven by cervical status and timing of cerclage placement rather than by increasing the number of sutures	

## Introduction

Cervical insufficiency is a multifactorial clinical condition in which the cervix undergoes painless dilation and effacement during the late second or early third trimester, primarily due to structural weakness or functional deficiency of the cervical tissue [1]. This premature cervical opening can lead to mid-trimester pregnancy loss, preterm premature rupture of membranes (PPROM), and spontaneous preterm birth, which collectively account for a considerable proportion of perinatal morbidity and mortality [2–4]. Although the condition affects only approximately 1% of pregnancies, its consequences are profound, often resulting in late miscarriage or extreme prematurity [5, 6].

Transvaginal cervical cerclage remains the standard surgical intervention for women at risk of cervical insufficiency. The procedure involves placing a circumferential suture at the cervicovaginal junction to provide mechanical reinforcement and reduce the risk of preterm birth [7]. Cerclage can be performed electively in women with a history of mid-trimester loss or an ultrasound-detected short cervix, or as an emergency (“rescue”) procedure when painless cervical dilation and bulging membranes are identified in the absence of labor or infection [3]. In such emergency scenarios, generally before 24 weeks of gestation and with ≥ 2 cm cervical dilation, prompt cerclage placement may avert pregnancy loss at a critical viability threshold [3]. However, in routine practice, the decision-making around technique and the degree of reinforcement are frequently individualized, and often influenced by baseline severity at presentation, which complicates interpretation of unadjusted comparisons between procedural approaches.

The McDonald technique, first described in 1957, remains the most widely adopted approach and traditionally utilizes a single purse-string suture placed with multiple passes through the cervical stroma [8]. Alternative modifications, such as the Wurm or double-stitch technique, employ two concentric sutures to provide potentially greater reinforcement [9]. However, whether the addition of a second suture truly improves obstetric outcomes remains uncertain. While some observational studies have suggested a potential benefit of double-suture cerclage in women with markedly shortened cervical length, others have failed to confirm a consistent advantage, and evidence from randomized or well-controlled studies remains limited and heterogeneous, particularly in emergency cerclage cases [10–14]. In routine clinical practice, the choice of cerclage configuration is rarely randomized and is often influenced by severity of cervical findings at presentation, such as cervical dilation or membrane prolapse. Consequently, selection bias and baseline imbalance represent major challenges in interpreting existing observational data. In the absence of clear consensus, the present study aimed to evaluate whether single versus double monofilament transvaginal cerclage is associated with differences in obstetric and neonatal outcomes under fully standardized surgical conditions, encompassing both prophylactic and emergency indications.

## Methods

### Study design and surgical technique

This single-center retrospective cohort study was conducted between 2020 and 2024 at Acıbadem Atakent Hospital, İstanbul, Turkey. All procedures were performed by a single, experienced surgical team using identical non-absorbable monofilament sutures and standardized perioperative protocols.

Women diagnosed with cervical insufficiency and treated with either prophylactic or emergency cerclage were included. Prophylactic cerclage was performed electively in asymptomatic women with a prior history of mid-trimester loss, spontaneous preterm birth, or those demonstrating significant cervical shortening (< 25 mm) on transvaginal ultrasound before 24 weeks. Emergency cerclage was performed in cases with painless cervical dilation or visible membranes in the absence of labor, rupture of membranes, or clinical infection.

Cervical length was measured transvaginally according to the Society for Maternal–Fetal Medicine (SMFM) guidelines [15]. A cervical length ≥ 30 mm was considered normal, 25–29 mm borderline, < 25 mm short (associated with increased preterm birth risk), and < 15 mm very short, typically warranting urgent clinical evaluation. These thresholds were used to guide prophylactic and emergency cerclage decisions in this cohort.

Women were excluded if they had active uterine contractions, vaginal bleeding, intra-amniotic infection, ruptured membranes, or major fetal anomalies at the time of evaluation. All patients received perioperative tocolysis and prophylactic antibiotics in accordance with institutional protocols.

All procedures were performed under general anesthesia with the patient in the dorsal lithotomy position. After establishing a sterile field, a weighted vaginal speculum was inserted to expose the cervix, which was gently grasped at the anterior lip with atraumatic forceps for stabilization. The bladder was emptied using a Foley catheter prior to suture placement.

In the single-suture (McDonald-type) cerclage, a monofilament non-absorbable suture (Prolene 1–0, Ethicon, USA) was placed circumferentially at the cervicovaginal junction with four equidistant passes at approximately the 2, 4, 8, and 10 o’clock positions, avoiding the cervical canal and maintaining a subepithelial depth of 5–7 mm. The knot was tied anteriorly at the 12 o’clock position with moderate tension sufficient to approximate the internal os without tissue strangulation. In the double-suture (modified Wurm-type) cerclage, a second identical monofilament stitch was placed 5–8 mm superior to the first. Both knots were tied anteriorly at the 12 o’clock position.

Because the surgical approach was not randomized, this study was analyzed as an observational comparison. Early in the study period, single-suture cerclage was the standard technique, and a second reinforcing stitch was gradually adopted over time as surgical practice evolved. Thus, the choice of suture number largely reflected temporal changes in operative technique rather than a predefined protocol based on cervical severity or individual surgeon preference.

After suture placement, hemostasis was confirmed and the cervix was irrigated with sterile saline. Perioperative antibiotic prophylaxis consisted of intravenous cefazolin (1 g) administered preoperatively and continued for 24 h. Vaginal progesterone (200 mg daily) was prescribed postoperatively until 36 weeks of gestation. All patients underwent transvaginal ultrasound within 24 h after the procedure to confirm fetal viability, verify correct suture placement, and measure post-cerclage cervical length. The mean operative time was approximately 20 ± 5 min, and estimated blood loss did not exceed 20 mL in any case.

The exposure of interest was the number of sutures placed during cerclage:

1. Single-suture group—McDonald-type cerclage using one monofilament stitch.
2. Double-suture group—modified Wurm-type cerclage using two concentric monofilament stitches.

### Variables, data collection, and outcomes

All clinical and perinatal data were retrospectively extracted from the hospital’s electronic medical record system by two independent reviewers. Data accuracy and completeness were verified through random cross-checking of selected cases to ensure consistency. The collected variables were categorized into four main domains. The full de-identified dataset used for the analyses is provided as Supplementary Table S1.

- Demographic parameters: maternal age, body mass index (BMI), gravidity, parity, number of living children, number of abortions, prior cervical surgery, prior preterm birth, LLETZ/conization history, pregnancy status (singleton or twin), and conception method (spontaneous or assisted reproductive technology).
- Perioperative parameters: gestational age at cerclage placement, pre-cerclage and post-cerclage cervical length, presence of cervical funneling, cervical dilation at diagnosis, presence of prolapsed membranes beyond the external os, and perioperative complications.
- Obstetric variables: gestational age at delivery (categorized as ≥ 37 weeks = term, < 37 weeks = preterm), latency period (days from cerclage to delivery), mode of delivery, and maternal complications (chorioamnionitis, atonia, retained placenta).
- Neonatal variables: birthweight, Apgar scores at 1 and 5 min, neonatal intensive care unit (NICU) admission, and neonatal mortality. Neonatal outcomes were recorded at the pregnancy level, with twin pregnancies classified as affected if at least one neonate met the outcome criteria. Because neonatal outcomes were analyzed at the pregnancy level rather than the individual neonate level, each pregnancy contributed a single observation to the analysis. This approach avoids intrapregnancy correlation between twins and therefore does not require additional clustering methods such as generalized estimating equations or mixed-effects models.

The primary outcome was term delivery, defined as delivery at ≥37+0 weeks’ gestation. Secondary obstetric outcomes included gestational age at delivery, latency period from cerclage placement to delivery, mode of delivery, and maternal complications, including chorioamnionitis, uterine atony, and retained placenta. Secondary neonatal outcomes included birthweight, Apgar scores at 1 and 5 minutes, NICU admission, and neonatal mortality.

### Statistical analysis

Baseline maternal demographic and obstetric characteristics were summarized according to cerclage suture number. Continuous variables are reported as mean ± standard deviation or median (interquartile range), as appropriate, and categorical variables as frequencies and percentages. Between-group comparisons were performed using the Mann–Whitney U test for continuous variables and Fisher’s exact test for categorical variables. *P *values are reported descriptively. Baseline comparability was assessed using standardized mean differences (SMDs), with values > 0.10 indicating meaningful imbalance. Perioperative variables were summarized descriptively and were not considered indicators of baseline comparability because they may reflect procedural or post-treatment factors.

Given the non-randomized allocation of single versus double cerclage sutures and the marked imbalance in baseline severity between groups, propensity score-based methods were used to mitigate confounding by indication. Propensity score weighting was selected rather than conventional multivariable regression due to the substantial baseline imbalance and the relatively small size of the single-suture cohort. This approach generates a weighted pseudo-population in which measured baseline covariates are more comparable between treatment groups, thereby improving causal interpretation in observational analysis.

Propensity scores estimating the probability of receiving double-suture cerclage were derived from logistic regression models including relevant pre-treatment maternal, obstetric, and cervical variables. Stabilized inverse probability of treatment weighting (IPTW) was applied, and extreme weights were truncated at the 1 st and 99th percentiles to reduce the influence of outliers. Covariate balance before and after weighting was evaluated using SMDs, with values < 0.10 considered indicative of acceptable balance. Balance diagnostics for pre-treatment covariates included in and assessed alongside the propensity score model are presented in Supplementary Table S2. Post-cerclage cervical length was not included in these diagnostics because it represents a post-treatment measurement.

For the primary outcome of term delivery (≥ 37 weeks), unadjusted risk ratios were first calculated. This was followed by a doubly robust IPTW-adjusted analysis, combining propensity score weighting with outcome modeling to further reduce potential residual confounding. Effect estimates are reported as risk ratios (RRs) with 95% confidence intervals (CIs). Secondary obstetric outcomes were summarized descriptively. For selected pregnancy-level neonatal outcomes, adjusted RRs were estimated when event numbers permitted. Because the unit of analysis was the pregnancy, each twin gestation contributed a single observation, thereby avoiding intrapregnancy clustering.

An exploratory multivariable logistic regression analysis was conducted to examine factors associated with term delivery, with results reported as adjusted odds ratios (ORs) and 95% CIs for descriptive purposes only. All analyses and graphical outputs were performed using R version 4.5.1 (R Foundation for Statistical Computing, Vienna, Austria) and GraphPad Prism version 10.0 (GraphPad Software, San Diego, CA, USA). Emphasis was placed on effect estimates and confidence intervals rather than statistical significance alone.

## Results

A total of 125 pregnant women who underwent transvaginal cervical cerclage during the study period were included in the analysis. Of these, 23 women (18.4%) received a single-suture cerclage and 102 women (81.6%) received a double-suture cerclage.

Baseline maternal demographic and obstetric characteristics according to cerclage suture number are presented in Table 1. Before propensity weighting, several baseline variables demonstrated numerical imbalance between groups, including gestational age at cerclage placement, prior preterm birth, twin pregnancy, prior cervical surgery, and conception method. After application of stabilized IPTW, covariate balance improved substantially across pre-treatment variables, with all post-weighting SMDs below 0.10 except BMI, which remained marginally above the threshold (Supplementary Table S2).

**Table 1:** Demographic characteristics by suture number

Variable	Single suture (*n* = 23)	Double suture (*n* = 102)	*p *value	SMD
Age (years)	33.0 ± 4.6 [22–40]	32.4 ± 4.3 [22–46]	0.383^a^	0.14
BMI (kg/m^2^)	29.4 ± 5.8 [20–40]	28.6 ± 5.1 [19–47]	0.624^a^	0.15
Gravidity	1 [1, 2]	1 [1–3]	0.398^a^	0.07
Parity	0 [0–0]	0 [0–0]	0.504^a^	0.00
Living children	0 [0–0]	0 [0–0]	0.617^a^	0.00
Number of prior miscarriages	0 [0–0]	0 [0–1]	0.671^a^	0.10
Pregnancy status				
Singleton pregnancy, n (%)	19 (82.6%)	77 (75.5%)	0.590^b^	0.18
Twin pregnancy, n (%)	4 (17.4%)	25 (24.5%)	–	–
Conception status				
Spontaneous conception, n (%)	13 (56.5%)	66 (64.7%)	0.481^b^	0.17
ART conception, n (%)	10 (43.5%)	36 (35.3%)	–	–
Prior cervical surgery, n (%)	2 (8.7%)	15 (14.7%)	0.736^b^	0.19
Prior preterm birth, n (%)	2 (8.7%)	21 (20.6%)	0.242^b^	0.34
LLETZ/Conization, n (%)	1 (4.3%)	9 (8.8%)	0.687^b^	0.18

*BMI* body mass index, *ART* assisted reproductive technology, *LLETZ* large loop excision of the transformation zone

Maternal demographic and obstetric history variables are summarized according to the number of sutures used for emergency cerclage (single vs. double)

Data are presented as mean ± standard deviation (SD) [range], median [IQR], or n (%)

Standardized mean differences (SMDs) were calculated to assess baseline imbalance; values > 0.10 indicate meaningful imbalance

^a^Mann–Whitney U test

^b^Fisher’s exact test. *p *values are descriptive

Maternal age and BMI were similar between groups (age: 33.0 ± 4.6 vs 32.4 ± 4.3 years; BMI: 29.4 ± 5.8 vs 28.6 ± 5.1 kg/m^2^). Twin pregnancies were present in 4 of 23 pregnancies (17.4%) in the single-suture group and 25 of 102 pregnancies (24.5%) in the double-suture group. A history of prior preterm birth and twin gestation were numerically more frequent in the double-suture group (20.6% vs. 8.7% and 24.5% vs. 17.4%, respectively).

Perioperative characteristics at the time of cerclage placement are summarized in Table 2. Double-suture cerclage was placed at a significantly earlier gestational age compared with single-suture cerclage (19.25 ± 4.12 vs. 21.51 ± 3.48 weeks; *p* = 0.011). Pre-cerclage cervical length was similar between groups (19.47 ± 8.66 vs. 19.52 ± 7.22 mm). Post-cerclage cervical length differed modestly between groups (median [IQR]: 23.0 [16.0–25.7] mm in the single-suture group vs. 23.0 [17.0–31.7] mm in the double-suture group; *p* = 0.024). Rates of cervical funneling (54.9% vs. 56.5%), cervical dilation (25.5% vs. 26.1%), and prolapsed membranes (14.7% vs. 21.7%) were similar between groups.

**Table 2:** Perioperative characteristics according to suture number

Variable	Single suture (*n* = 23)	Double suture (*n* = 102)	*p *value
Gestational age at cerclage (weeks)	21.51 ± 3.48 [13.43–26.00]	19.25 ± 4.12 [12.43–30.14]	**0.011**^**a**^
Pre-cerclage cervical length (mm)	19.52 ± 7.22 [3.00–32.00]	19.47 ± 8.66 [2.00–39.90]	0.735^a^
Post-cerclage cervical length (mm)	23 [16.0–25.7]	23 [17.0–31.7]	**0.024**^a^
Pre-cerclage funneling, n (%)	13 (56.5%)	56 (54.9%)	1.000^b^
Cervical dilation at diagnosis, n (%)	6 (26.1%)	26 (25.5%)	1.000^b^
Prolapsed membranes beyond external os, n (%)	5 (21.7%)	15 (14.7%)	0.528^b^
Perioperative complications, n (%)	0 (0.0%)	4 (3.9%)	1.000^b^

Bold values indicate statistically significant differences between groups (p < 0.05)

Perioperative parameters at the time of cerclage placement are presented for single- and double-suture groups

Data are presented as mean ± standard deviation (SD) [range], median [IQR], or n(%)

^a^Mann–Whitney U test

^b^Fisher’s exact test

**Fig. 1:**
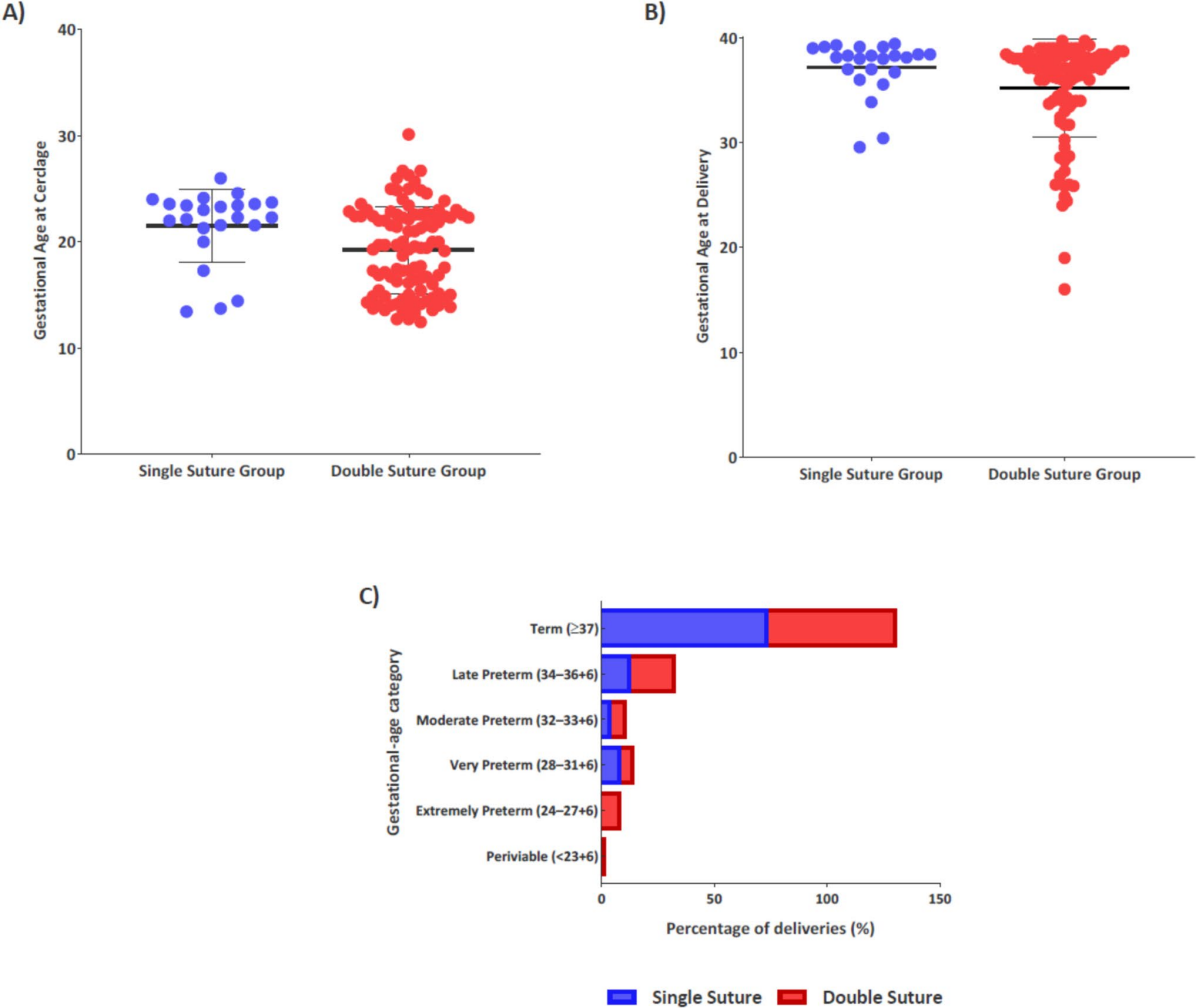
Obstetric outcomes following single- versus double-suture cervical cerclage. **A** Gestational age at cerclage placement according to suture type. **B** Gestational age at delivery according to suture type. **C** Distribution of deliveries by gestational-age category (term, late preterm, moderate preterm, very preterm, extremely preterm, periviable)

Descriptive obstetric outcomes according to cerclage suture number are shown in Table 3. Mean gestational age at delivery and latency from cerclage placement to delivery were comparable between groups. The proportion of pregnancies reaching term (≥ 37 weeks) was numerically higher in the single-suture group (73.9% vs. 56.9%) although this difference was not statistically significant. Mode of delivery and maternal complication rates did not differ meaningfully between groups. The distribution of gestational age at cerclage placement and delivery according to suture number is illustrated in Fig. 1.

**Table 3:** Obstetric outcomes according to suture number

Variable	Single suture (n = 23)	Double suture (*n* = 102)	*p *value
Gestational age at delivery, n (%)			
Term (≥ 37 + 0 weeks)	17 (73.9%)	58 (56.9%)	0.161^b^
Preterm (< 37 weeks)	6 (26.1%)	44 (43.1%)	0.161^b^
Late preterm (34 + 0–36 + 6)	3 (13.0%)	20 (19.6%)	0.565^b^
Moderate preterm (32 + 0–33 + 6)	1 (4.3%)	6 (5.9%)	1.000^b^
Very preterm (28 + 0–31 + 6)	2 (8.7%)	7 (6.9%)	0.670^b^
Extremely preterm (24 + 0–27 + 6)	0 (0.0%)	9 (8.8%)	0.187^b^
Periviable (≤ 23 + 6)	0 (0.0%)	2 (2.0%)	1.000^b^
Latency period from cerclage placement to delivery (days)	109.7 ± 31.3 [25–173]	111.8 ± 42.2 [11–175]	0.676^a^
Delivery mode, n (%)			
Vaginal delivery	13 (56.5%)	46 (45.1%)	0.371^b^
Cesarean section	10 (43.5%)	56 (54.9%)	—
Maternal complications, n (%)			
Chorioamnionitis	1 (4.3%)	1 (1.0%)	0.335^b^
Atonia	0 (0.0%)	2 (2.0%)	1.000^b^
Retained placenta	0 (0.0%)	3 (2.9%)	1.000^b^

Adjusted association between suture number and term delivery (≥ 37 weeks)			
Analysis	Effect estimate (RR)	95% CI	*p *value
Unadjusted	0.77	0.57–1.05	0.16
IPTW-adjusted	0.89	0.73–1.09	0.24
Doubly robust IPTW-adjusted	0.87	0.71–1.07	0.18

Descriptive obstetric outcomes according to single- and double-suture cerclage, including gestational age at cerclage and delivery, latency period, delivery mode, and maternal complications. The adjusted association between suture number and term delivery (≥ 37 weeks) is presented at the bottom of the table using propensity score-based methods

Data are presented as mean ± standard deviation (SD) [range] for continuous variables and n (%) for categorical variables

^a^Mann–Whitney U test

^b^Fisher’s exact test. For descriptive obstetric outcomes, *p* values are unadjusted and descriptive, reflecting differences in clinical presentation and perioperative context. Subcategories of preterm birth are shown for clinical description only and were not interpreted as independent inferential comparisons

In unadjusted analysis, double-suture cerclage was not associated with a statistically significant difference in the likelihood of term delivery (RR 0.77, 95% CI 0.57–1.05). After adjustment using IPTW, this association remained attenuated (IPTW-adjusted RR 0.89, 95% CI 0.73–1.09). In the primary doubly robust IPTW-adjusted analysis, no statistically significant difference in term delivery was observed between double- and single-suture cerclage (aRR 0.87, 95% CI 0.71–1.07).

Sensitivity analysis restricted to singleton pregnancies and stratified by cerclage indication (prophylactic vs emergency) yielded results consistent with the primary analysis. Similarly, neonatal outcomes restricted to singleton pregnancies did not differ significantly between groups (Supplementary Table S3).

Pregnancy-level neonatal outcomes according to cerclage suture number are summarized in Table 4. Mean pregnancy-level birthweight was numerically higher in the single-suture group. In adjusted pregnancy-level analyses, no statistically detectable differences were observed between groups for NICU admission (aRR 1.10, 95% CI 0.74–1.63) or Apgar score < 7 at 5 min (aRR 1.08, 95% CI 0.69–1.71). Neonatal mortality events were rare and therefore precluded reliable adjusted estimation.

**Table 4:** Pregnancy-level neonatal outcomes according to cerclage suture number, including descriptive outcomes and adjusted associations

Descriptive neonatal outcomes (per pregnancy)			
Neonatal variable	Single suture (*n* = 23 pregnancies)	Double suture (*n* = 102 pregnancies)	
Mean birthweight (g)	2909 ± 736	2532 ± 809	
Any Apgar score < 7 at 1 min, n (%)	5 (21.7%)	33 (32.4%)	
Any Apgar score < 7 at 5 min, n (%)	3 (13.0%)	21 (20.6%)	
Any NICU admission, n (%)	4 (17.4%)	31 (30.4%)	
Any neonatal death, n (%)	0 (0.0%)	5 (4.9%)	

Adjusted association between suture number and pregnancy-level neonatal outcomes			
Outcome (per pregnancy)	Single suture (*n* = 23)	Double suture (*n* = 102)	Adjusted RR (95% CI)
Any NICU admission	4 (17.4%)	31 (30.4%)	1.10 (0.74–1.63)
Any Apgar score < 7 at 5 min	3 (13.0%)	21 (20.6%)	1.08 (0.69–1.71)
Any neonatal death	0 (0.0%)	5 (4.9%)	—

Data are presented as mean ± standard deviation (SD) [range] for continuous variables and n (%) for categorical variables

**Fig. 2:**
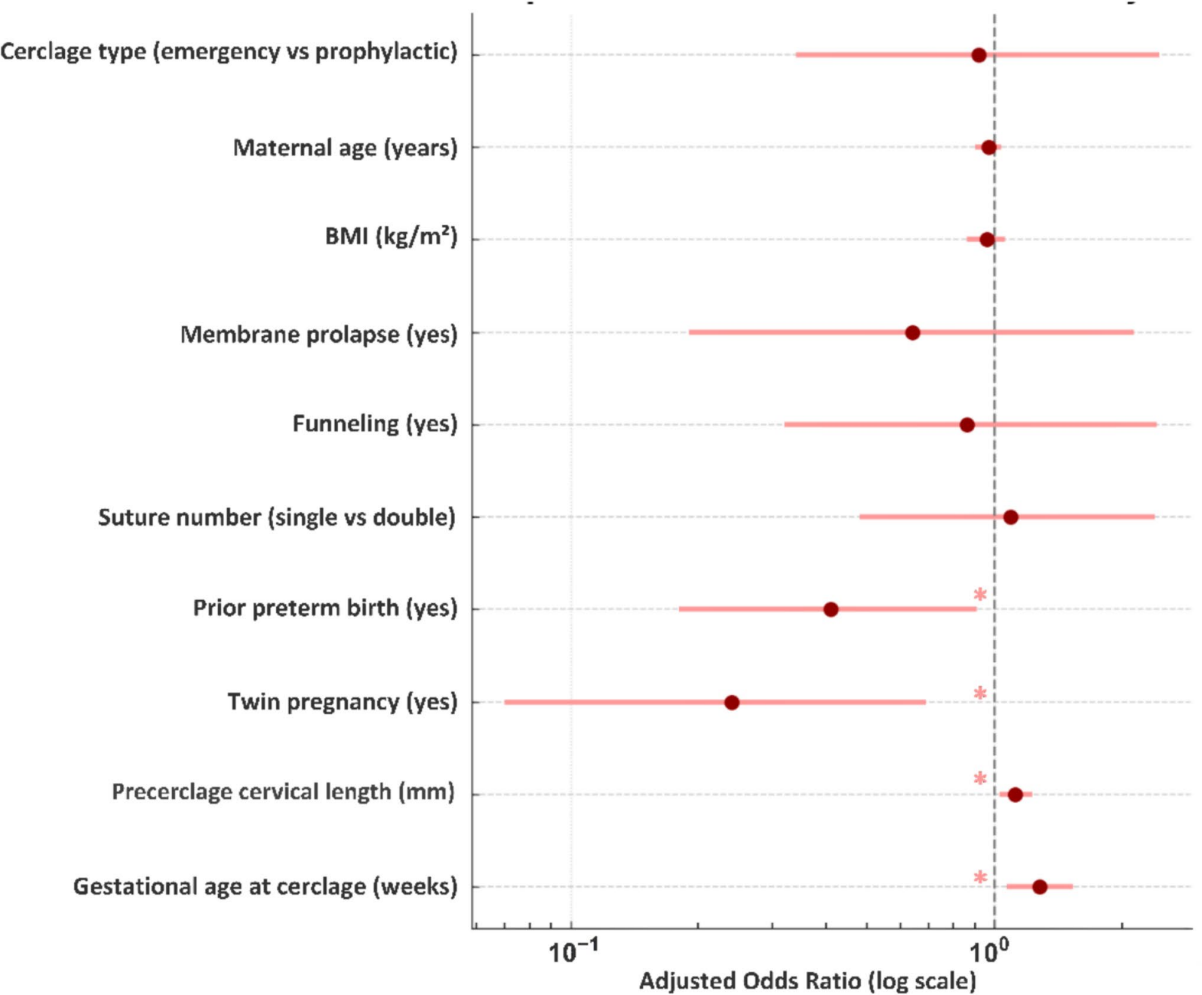
Exploratory multivariable analysis of factors associated with term delivery (≥ 37 weeks) following cervical cerclage

In exploratory multivariable logistic regression model, gestational age at cerclage placement and pre-cerclage cervical length were positively associated with term delivery, whereas twin pregnancy and prior preterm birth were negatively associated (Fig. 2). Cerclage suture number was not independently associated with term delivery (aOR 1.09, 95% CI 0.48–2.39).

Overall, across both descriptive analysis and propensity-adjusted models, cerclage suture number was not independently associated with term delivery or pregnancy-level neonatal outcomes.

## Discussion

This study evaluated whether the number of sutures used during transvaginal cervical cerclage influences obstetric and neonatal outcomes under standardized surgical conditions. By employing a single experienced surgical team, identical monofilament suture material and uniform perioperative protocols, the analysis aimed to isolate suture number as a technical factor while minimizing operator- and material-related variability.

In this cohort, double-suture cerclage appeared more frequently in later years of the study period as surgical practice evolved. Early in the study period, a single McDonald-type cerclage represented the standard technique, whereas the addition of a second reinforcing stitch was gradually adopted over time. As a result, the distribution of suture number partly reflects temporal changes in operative practice rather than a strictly predefined protocol based on cervical severity at presentation. Despite these baseline differences, both single-suture (McDonald-type) and double-suture (modified Wurm-type) techniques were associated with effective pregnancy prolongation, with similar latency periods and gestational ages at delivery. After propensity score-based adjustment accounting for confounding by indication, suture number was not independently associated with term delivery or pregnancy-level neonatal outcomes. Importantly, the confidence interval surrounding the adjusted relative risk (0.71–1.07) includes the possibility of modest benefit or harm, indicating that clinically meaningful differences between techniques cannot be completely excluded. Within the precision of this cohort, however, we did not observe a statistically significant improvement in outcomes with the addition of a second suture compared with a single, well-positioned stitch.

The influence of suture number on cerclage efficacy remains debated [10–14]. Several previous studies have examined differences in cerclage configuration although many have simultaneously varied surgical technique or suture material, making it difficult to isolate the mechanical contribution of multiple sutures [7, 16, 17]. Donadono et al. reported improved outcomes with double-suture cerclage among women with markedly shortened cervices, whereas other observational and controlled studies have generally found no consistent differences in pregnancy prolongation or neonatal outcomes between single- and double-suture approaches [10, 12, 13]. Xu et al. observed superior outcomes with double sutures in emergency cerclage; however, this finding may partly reflect selection bias as more severe cases often receive reinforced cerclage in clinical practice [18].

Our findings are consistent with this body of evidence. In the present cohort, women receiving double-suture cerclage more frequently exhibited markers of advanced cervical insufficiency, including earlier gestational age at cerclage placement and higher baseline obstetric risk. Exploratory multivariable analysis further demonstrated that indicators of cervical status at the time of intervention—such as gestational age at cerclage placement and pre-cerclage cervical length—were associated with the likelihood of term delivery, whereas the number of sutures was not. These observations reinforce the concept that cervical condition at presentation, rather than the number of sutures applied, is the primary determinant of successful pregnancy prolongation [19–24].

One possible explanation relates to the underlying biology of cervical insufficiency. The condition often reflects structural alterations in cervical connective tissue, including collagen remodeling, inflammatory activation, and reduced tissue integrity. When cervical tissue has already undergone significant shortening or dilation, the mechanical strength of the cervical stroma may become the limiting factor rather than the number of reinforcing sutures. Because both sutures anchor within the same compromised cervical tissue, placement of an additional stitch may provide limited incremental mechanical support once cervical remodeling has progressed.

The inclusion of both prophylactic and emergency cerclage cases enhances the clinical relevance and generalizability of these findings. After adjustment for baseline heterogeneity, outcomes were consistent across suture groups, suggesting that stitch number alone does not meaningfully alter prognosis when surgical timing, technique, and perioperative management are optimized. These findings also align with evidence from the UK C-STICH trial, which compared monofilament and braided suture materials for cervical cerclage and demonstrated no significant difference in pregnancy loss, although monofilament sutures were associated with a lower risk of infection. In the present study, all cerclage procedures were performed using identical monofilament sutures, thereby eliminating variability related to suture material and allowing the analysis to more specifically focus on the potential effect of suture number [17, 25]. By standardizing suture material across all procedures, our findings suggest that differences in outcomes are more likely related to baseline cervical condition rather than technical variations in cerclage configuration.

Neonatal outcomes also appeared to be influenced more by underlying pregnancy characteristics than by cerclage configuration. Pregnancy-level neonatal outcomes, including birthweight, Apgar scores, NICU admission, and neonatal mortality, did not differ significantly by suture number after adjustment. As expected, plurality remained an important determinant of neonatal risk, consistent with established evidence that multifetal gestations inherently carry a higher risk of prematurity and adverse neonatal outcomes [26, 27].

More broadly, the effectiveness of cervical cerclage is shaped by several factors beyond suture number, including timing of placement, residual cervical length, degree of cervical dilation, and membrane prolapse at presentation [28–33]. Earlier intervention before advanced cervical effacement or membrane exposure has been associated with improved latency and neonatal survival. Operator experience, appropriate suture tension, and adjunctive therapies, such as progesterone, tocolytics, and antibiotics, may also contribute to procedural success [34]. Emerging evidence also highlights the role of cervical tissue biology, including alterations in collagen cross-linking, inflammatory cytokine balance, and matrix metalloproteinase activity, in influencing cervical remodeling and the mechanical response to cerclage [35, 36].

The strengths of this study include its standardized surgical approach, consistent perioperative management, and the use of propensity score-based methods to address confounding by indication. Nevertheless, several limitations should be considered. The retrospective design introduces the possibility of residual confounding despite statistical adjustment. Although propensity score-based IPTW substantially improved baseline comparability, a small degree of imbalance remained for BMI after weighting. Additionally, the two study groups were unequal in size, with a relatively small number of patients in the single-suture cohort compared with the double-suture cohort. This imbalance partly reflects temporal changes in surgical practice at our center, where a second reinforcing stitch was gradually adopted over time rather than being applied according to a predefined protocol. Although IPTW and doubly robust models were used to mitigate confounding, non-random treatment allocation may still introduce residual bias.

Furthermore, the relatively small sample size, particularly within the single-suture group, may limit statistical power and the precision of effect estimates. Therefore, the absence of statistically significant differences should not be interpreted as definitive evidence of equivalence between techniques. Finally, the single-center setting may limit generalizability to institutions with different patient populations or surgical practices. Future prospective studies and randomized trials are needed to further clarify whether specific high-risk populations may benefit from alternative cerclage configurations.

## Conclusion

Under standardized operative conditions and after accounting for baseline clinical severity, the number of sutures used during transvaginal cervical cerclage was not associated with improved obstetric or neonatal outcomes. These findings suggest that a single, well-placed monofilament suture may be sufficient in many clinical settings. Larger prospective or randomized studies are required to more definitively determine whether suture number influences outcomes.Overall, cervical status at presentation and timing of intervention are more important clinical determinants of outcome than minor technical variations in cerclage configuration.

## Supplementary Information

Below is the link to the electronic supplementary material.

Supplementary file1 (XLSX 35 KB)

Supplementary file2 (XLSX 9 KB)

Supplementary file3 (XLSX 10 KB)

## Data Availability

The data supporting the findings of this study are available within the article and its supplementary materials.
